# Long Non-Coding RNA HOXA Transcript Antisense RNA Myeloid-Specific 1–HOXA1 Axis Downregulates the Immunosuppressive Activity of Myeloid-Derived Suppressor Cells in Lung Cancer

**DOI:** 10.3389/fimmu.2018.00473

**Published:** 2018-03-12

**Authors:** Xinyu Tian, Jie Ma, Ting Wang, Jie Tian, Yue Zhang, Lingxiang Mao, Huaxi Xu, Shengjun Wang

**Affiliations:** ^1^Department of Laboratory Medicine, The Affiliated People’s Hospital, Jiangsu University, Zhenjiang, China; ^2^Department of Immunology, Jiangsu Key Laboratory of Laboratory Medicine, School of Medicine, Jiangsu University, Zhenjiang, China; ^3^Department of Laboratory Medicine, Jiangsu Cancer Hospital, Nanjing, China

**Keywords:** long non-coding RNA, HOXA transcript antisense RNA myeloid-specific 1, HOXA1, myeloid-derived suppressor cells, lung cancer

## Abstract

HOXA transcript antisense RNA myeloid-specific 1 (HOTAIRM1) is a long non-coding RNA that has been shown to be a key regulator of myeloid cell development by targeting HOXA1. Myeloid-derived suppressor cells (MDSCs) are a heterogeneous population of immature myeloid cells that possess immunosuppressive function. However, the impact of HOTAIRM1 on the development of MDSCs remains unknown. In this study, we demonstrated that HOTAIRM1 was expressed in MDSCs and that overexpression of HOTAIRM1 could downregulate the expression of suppressive molecules in MDSCs. In addition, HOTAIRM1 levels were observed to be decreased in the peripheral blood cells of lung cancer patients compared with those of healthy controls. By analyzing HOTAIRM1 expression levels in different types of lung cancer, we found that HOTAIRM1 was mainly expressed in lung adenocarcinoma. Finally, it was confirmed that HOTAIRM1 could enhance the expression of HOXA1 in MDSCs and that high levels of HOXA1, the target gene of HOTAIRM1, could delay tumor progression and enhance the antitumor immune response by downregulating the immunosuppression of MDSCs. Taken together, this study illustrates that HOTAIRM1/HOXA1 downregulates the immunosuppressive function of MDSCs and may be a potential therapeutic target in lung cancer.

## Introduction

Lung cancer has become the most commonly diagnosed cancer worldwide. Lung cancer can be divided into non-small cell lung cancer, which includes adenocarcinoma (ADC) and squamous cell carcinoma, and small cell lung cancer. Despite improvements in chemotherapy and the integration of lung cancer-targeted therapy, the 5-year survival rate is no higher than 15% after the initial diagnosis (1–4). Immunotherapy has recently been drawing increasing attention as a lung cancer therapy (5–8). Thus, antitumor therapy by targeting myeloid-derived suppressor cells (MDSCs), which are a main cause of tumor escape, is attracting increased interest (9).

Myeloid-derived suppressor cells are a population of heterogeneous cells derived from bone marrow. Under physiological conditions, these immature myeloid cells can immediately differentiate into mature granulocytes, monocytes and macrophages. However, under pathological conditions, especially cancer, MDSC differentiation is blocked, which leads to the expansion of MDSCs (10–13). In both humans and mice, MDSCs can be divided into polymorphonuclear MDSCs (PMN-MDSCs) and monocytic MDSCs (M-MDSCs) (14–17). In humans, MDSCs represent a population of cells with the CD11b^+^CD33^+^HLA-DR^−^CD14^−^ phenotype and are further distinguished into PMN-MDSCs and M-MDSCs based on the different expression of Lin and CD15 (18, 19). In cancer development, MDSCs accelerate tumor progression by inhibiting the T cell response and inducing regulatory T cells by releasing arginase 1 (Arg1), reactive oxygen species (ROS), and inducible nitric oxide synthase. Currently, therapies targeting MDSCs mainly involve promoting the differentiation of MDSCs, inhibiting the suppressive effect of MDSCs or eliminating MDSCs (19–21).

Long non-coding RNAs (lncRNAs) are transcripts >200 nt in length that do not encode proteins. Compared with mRNAs, lncRNAs contain fewer exons and are less conserved among different species. In addition, the expression of lncRNAs is cell- and stage specific (22, 23). Increasing evidence has indicated that lncRNAs are involved in modulating cellular biology *via* various mechanisms (24–26). lncRNA HOXA transcript antisense RNA myeloid-specific 1 (HOTAIRM1), which is transcribed by RNA polymerase II, is located between the human HOXA1 and HOXA2 genes. HOTAIRM1, which shows myeloid-specific expression, is transcribed antisense to HOXA genes and originates from the same CpG island that embeds the start of HOXA1 (27). HOXA1 has been indicated as a crucial transcriptional regulator in definitive hematopoiesis. Moreover, functions of homeobox pathways in normal hematopoiesis and acute myeloid leukemia have been well documented. HOTAIRM1/HOXA1 has been shown to modulate myeloid cell differentiation (28). During granulocyte differentiation, HOTAIRM1 is the most prominently expressed intergenic transcript. Knockdown of HOTAIRM1 significantly inhibits the expression of HOXA1 and HOXA4 and reduces the induction of myeloid differentiation gene transcripts, such as CD11b and CD18, during RA-induced granulocytic differentiation (29–31). However, the role of HOTAIRM1/HOXA1 in the development and suppressive function of MDSCs remains unknown.

## Materials and Methods

### Patients and Samples

We collected 300 peripheral blood samples from lung cancer patients (lung ADC, *n* = 160; squamous cell lung cancer, *n* = 72; and small cell lung cancer, *n* = 68) and 285 peripheral blood samples from healthy donors. Peripheral blood was centrifuged at 20°C and 2,000 rpm for 5 min to separate the cells from plasma. Red cells were lysed by ACK, and the remaining cells were used in subsequent experiments. Paired peripheral blood samples were collected from 42 lung cancer patients before and after surgery. Paired lung cancer and cancer-adjacent normal tissues were collected from nine patients who underwent primary surgical resection of lung cancer. This study was carried out in accordance with the ethical standards of the Affiliated People’s Hospital of Jiangsu University with written informed consent from all subjects. All subjects provided written informed consent in accordance with the Declaration of Helsinki. The protocol was approved by the ethics committee of the Affiliated People’s Hospital with Jiangsu University.

### Isolation of MDSCs from Tumor Tissues of Lung Cancer Patients

Tumor tissues and adjacent tissues from lung cancer patients were cut into small pieces (1–2 mm^3^) and digested with collagenase II (Sigma-Aldrich, St. Louis, MO, USA) at 37°C for 2 h on a rotating platform to obtain single-cell suspensions. The obtained cells were stained with PE/Cy5-anti-mouse/human-CD11b (2.5 μg/10^6^ cells) (clone: M1/70), FITC-antihuman-CD33 (2.5 μg/10^6^ cells) (clone: HIM3–4), PE-antihuman-CD14 (2.5 μg/10^6^ cells) (clone: 61D3), and APC-anti-HLA-DR (2.5 μg/10^6^ cells) (clone: LN3) mAbs (eBioscience, San Diego, CA, USA) for 30 min. Then, cells were washed with PBS containing 2% FBS twice (4°C, 500 g, 5 min) and subjected to isolation with FCM (ARIAII, BD, USA). The purity of isolated MDSCs was greater than 85%.

### Induction of Human MDSCs

Human peripheral blood mononuclear cells (PBMCs) were isolated by density-gradient centrifugation using Ficoll-Hypaque solution as previously described (32). The PBMCs from healthy donors were seeded onto 6-well plates and stimulated with 40 ng/mL GM-CSF (Peprotech, NJ, USA) and 40 ng/mL IL-1β (Peprotech, NJ, USA) for 96 h. Then, cells were collected and purified with human anti-CD33 beads (Miltenyi Biotec, Auburn, CA, USA) following the manufacturer’s protocol. The purity of isolated MDSCs was greater than 85%.

### Flow Cytometry

To detect the proportion of Th1 and CTL cells, PBMCs from lung cancer patients and healthy donors were stimulated with 50 ng/mL phorbol myristate acetate (PMA; Sigma-Aldrich, CA, USA) and 1 µg/mL ionomycin (Sigma-Aldrich, CA, USA) for 2 h and then incubated for an additional 4 h in the presence of 1 µg/mL brefeldin-A (eBioscience, San Diego, CA, USA) at 37°C and 5% CO_2_. After that, cells were stained with human anti-CD3 and anti-CD8 mAbs (eBioscience), fixed, permeabilized, and stained with human anti-IFN-γ mAb (eBioscience) according to the Intracellular Staining Kit (Invitrogen, Carlsbad, CA, USA) instructions.

### RNA Isolation and Quantitative Real-time PCR

Total RNA was isolated from cells with TRIzol reagent (Invitrogen, CA, USA), according to the manufacturer’s instructions. The cDNA was synthesized with random primers and the ReverTraAca^®^qPCR RT kit (Toyobo, Osaka, Japan). Quantitative real-time PCR was performed as previously described (33). Primer sequences were as follows: human β-actin, sense 5-GAGTGTGGAGACCATCAAGGA-3, antisense 5-TGTATTGCTTTGCGTTGGAC-3; human HOTAIRM1, sense 5-AGGCCGATTTGGAGTGCT-3, antisense 5-TCTCGCCAGTTCATCTTTCA-3; human 18s, sense 5-CGGACAGGATTGACAGATTG-3, antisense 5-GCCAGAGTCTCGTTCGTTATC-3; human ARG1, sense 5-CCTTTGCTGACATCCCTAAT-3, antisense 5-GATTCTTCCGTTCTTCTTGACT-3; and human HOXA1, sense 5-TCACGGAACTGGAGAAGGAG-3, antisense 5-GGAGAGATGGGCAAGAGACC-3. The level of each gene was expressed as a ratio to the β-actin transcript level.

### Plasmids and Transfection

The MDSCs were seeded onto 48-well plates and then transfected with pcDNA3.1-HOTAIRM1, pcDNA3.1-HOXA1, or pcDNA3.1-vector (ThermoFisher, MA, USA) following the manufacturer’s protocol.

### Isolation of Murine MDSCs

The MDSCs isolated from the spleen and tumor tissue of tumor-bearing mice were isolated using a mouse MDSC isolation kit (Miltenyi Biotec, Auburn, CA, USA) following the manufacturer’s protocol. To isolate MDSCs from the spleen, the spleen of tumor-bearing mice was removed aseptically and then minced to obtain single-cell suspensions. The obtained cells were stained with anti-mouse-CD11b-beads (Miltenyi Biotec, Auburn, CA, USA) for 30 min and washed with PBS twice. Then, cells were subjected to isolation with a column. To isolate MDSCs from the tumor tissues of tumor-bearing mice, tumor tissues were cut into small pieces (1–2 mm^3^) and digested with collagenase II (Sigma-Aldrich, St. Louis, MO, USA) at 37°C for 2 h on a rotating platform to obtain single-cell suspensions. The obtained cells were stained with anti-mouse-CD11b-beads (Miltenyi Biotec, Auburn, CA, USA) for 30 min and washed with PBS twice. Then, cells were subjected to isolation with a column. The purity of MDSCs isolated from spleen and tumor tissues was greater than 90 and 80%, respectively.

### Detection of Arginase Activity and ROS Production

Arginase activity was detected with the QuantiChrom Arginase Assay kit (BioAssay Systems, Hayward, CA, USA) and computed following the manufacturer’s instructions.

The level of ROS produced by G-MDSCs was measured using the oxidation-sensitive dye 2,7-dichlorofluorescin diacetate (Invitrogen, Carlsbad, CA, USA). The G-MDSCs were harvested and cultured with PMA (30 ng/mL) and oxidation-sensitive dye 2,7-dichlorofluorescin diacetate (2.5 nM) for 0.5 h. After that, cells were detected by flow cytometry.

### *In Vivo* Experiments

To investigate whether HOXA1 could directly impair antitumor immune responses, 0.9 × 10^6^ LLC cells were transplanted into C57BL/6 mice. After a palpable tumor was formed, tumor-bearing mice were treated by intratumoural injections of pcDNA3.1-HOXA1 every 2–3 days for 3 weeks. Tumor volume and weight were measured, and single-cell suspensions from the spleen, draining lymph nodes and tumors of tumor-bearing mice were collected for analysis. Cells were stimulated with PMA (Sigma-Aldrich, St. Louis, MO, USA, 50 ng/mL), ionomycin (eBioscience, San Diego, CA, USA, 1 µg/mL), and monensin (eBioscience, 2 µg/mL) for 5 h. After that, cells were stained with anti-CD3 and anti-CD4/CD8 mAbs (eBioscience), fixed, permeabilized and stained with anti-IFNγ mAb (eBioscience) according to the Intracellular Staining Kit (Invitrogen, Carlsbad, CA, USA) instructions. Cells obtained from tumor tissues were stained with anti-CD11b and anti-Gr1 mAbs (BioLegend). All of the experiments were approved by the Animal Use Committee of Jiangsu University in accordance with the International Guiding Principles for Biomedical Research Involving Animals.

### Graphing and Statistical Analysis of Data

Data are presented as the mean ± SD. Data from all experiments were entered into GraphPad Prism5.0 (GraphPad, San Diego, CA, USA) to generate bar graphs or graphs of tumor regression. The statistical significance of differences between groups was determined by Student’s *t*-test. Correlations between variables were determined by Spearman’s correlation coefficient. Differences were considered significant at a *P* value less than 0.05.

## Results

### HOTAIRM1 Can Inhibit the Development of MDSCs in Lung Cancer

**Figure 1:**
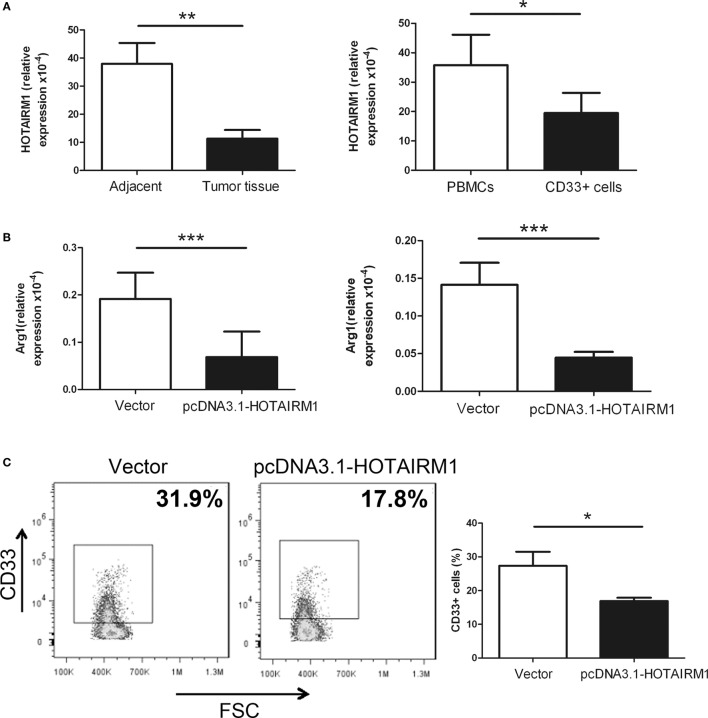
HOXA transcript antisense RNA myeloid-specific 1 (HOTAIRM1) can inhibit myeloid-derived suppressor cell (MDSC) development in lung cancer. **(A)** The expression level of HOTAIRM1 in MDSCs isolated from tumor tissues of patients with lung cancer (left) or induced from peripheral blood mononuclear cells (PBMCs) of healthy donors with GM-CSF (40 ng/mL) + IL-1β (40 ng/mL) (right) was detected with qRT-PCR. **(B)** The MDSCs isolated from tumor tissues of patients with lung cancer (left) or induced from PBMCs of healthy donors with GM-CSF (40 ng/mL) + IL-1β (40 ng/mL) (right) were transfected with pcDNA3.1-HOTAIRM1, and arginase 1 (Arg1) levels were detected by qRT-PCR. **(C)** The effect of HOTAIRM1 overexpression on the induction of MDSCs (***P < 0.001, **P < 0.01, and *P < 0.05).

To validate the influence of HOTAIRM1 on the immunosuppression of MDSCs, we first confirmed whether HOTAIRM1 is specifically expressed in MDSCs. Thus, we isolated CD11b^+^CD33^+^HLA-DR^−^CD14^−^ MDSCs from tumor tissues of lung cancer patients, and qRT-PCR was used to detect the level of HOTAIRM1. The expression level of HOTAIRM1 in MDSCs from tumor tissues was significantly decreased compared with that in cells with the same phenotype from adjacent tissue. We also detected the HOTAIRM1 expression level in MDSCs induced from PBMCs of healthy donors and found that the HOTAIRM1 expression level was decreased in the induced MDSCs compared with PBMCs without stimulation (Figure 1A). In addition, overexpression of HOTAIRM1 downregulated the Arg1 expression level in both isolated and induced MDSCs (Figure 1B). Surprisingly, during MDSC induction, overexpression of HOTAIRM1 could suppress the induction of CD33^+^ MDSCs (Figure 1C).

### The Proportion of MDSCs Is Negatively Correlated with the Percentage of Th1/CTL Cells in the Peripheral Blood of Lung Cancer Patients

**Figure 2:**
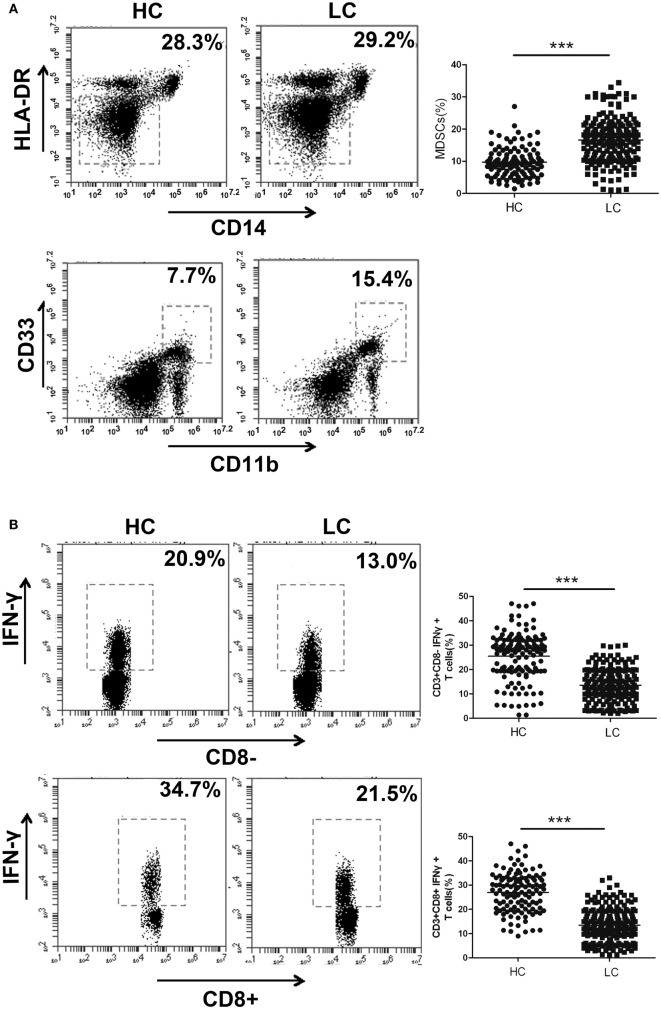

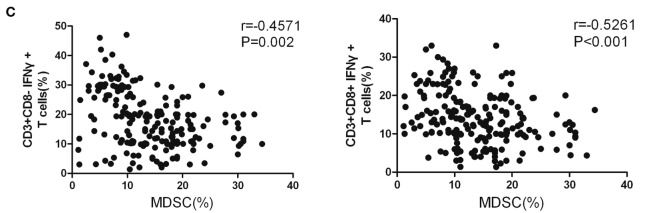
The proportion of myeloid-derived suppressor cells (MDSCs) is negatively correlated with the percentage of Th1/CTL cells in the peripheral blood of lung cancer patients. **(A)** The proportion of CD11b^+^CD33^+^HLA-DR^−^CD14^−^ MDSCs in the peripheral blood of healthy controls (*n* = 300) and lung cancer patients (*n* = 285). **(B)** The proportions of CD3^+^CD8-IFN-γ^+^ Th1 and CD3^+^CD8^+^IFN-γ^+^ CTL cells in the peripheral blood of healthy controls (*n* = 285) and patients with lung cancer (*n* = 300). **(C)** The correlation between the proportion of MDSCs and the percentage of CD3^+^CD8-IFN-γ^+^ Th1/CD3^+^CD8^+^IFN-γ^+^ CTL cells in the peripheral blood of patients with lung cancer (*n* = 300) (***P < 0.001).

In the following experiments, we aimed to confirm whether the expression level of HOTAIRM1 in the peripheral blood could be used to reflect the progression of lung cancer. First, we collected 300 peripheral blood samples from lung cancer patients and confirmed the immunosuppressive environment in lung cancer. The proportion of MDSCs in the peripheral blood of patients with lung cancer was observed to be much higher than that of healthy controls (Figure 2A). In addition, we found that the percentages of Th1 and CTL cells in the peripheral blood of patients with lung cancer were significantly decreased compared with those in the peripheral blood of healthy controls (Figure 2B). Correlation analysis indicated that the proportion of MDSCs was negatively correlated with the proportion of Th1/CTL cells (Figure 2C). These results demonstrated the immunosuppressive environment in patients with lung cancer.

### The Expression of HOTAIRM1 Was Significantly Decreased in the Peripheral Blood of Patients with Lung Cancer

**Figure 3:**
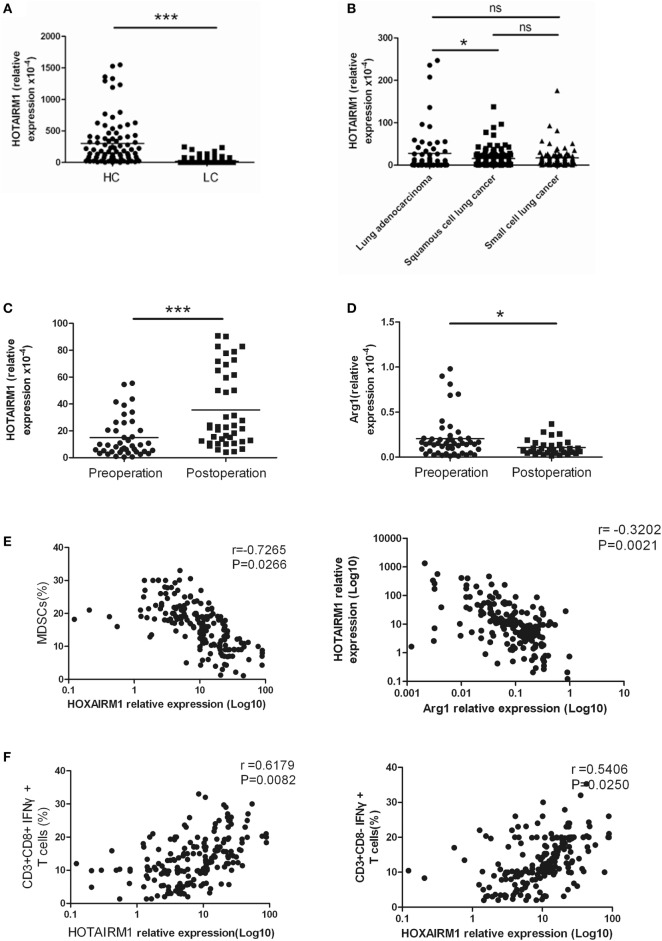
The expression of HOXA transcript antisense RNA myeloid-specific 1 (HOTAIRM1) was significantly decreased in the peripheral blood of patients with lung cancer. **(A)** The expression of HOTAIRM1 in the peripheral blood of lung cancer patients (*n* = 300). **(B)** The expression of HOTAIRM1 in the peripheral blood of patients with different types of lung cancer (*n* = 300). **(C)** HOTAIRM1 expression levels in the peripheral blood of lung cancer patients pre- and post-operation (*n* = 42). **(D)** Arginase 1 (Arg1) levels in the peripheral blood of lung cancer patients pre- and postoperation (*n* = 42). **(E)** Correlation analysis between HOTAIRM1 levels and the proportion of myeloid-derived suppressor cells (MDSCs) or the Arg1 levels in the peripheral blood of patients with lung cancer (*n* = 300). **(F)** The correlation between the expression of HOTAIRM1 and the proportion of CD3^+^CD8-IFN-γ^+^/CD3^+^CD8^+^IFN-γ^+^ cells in the peripheral blood of lung cancer patients (*n* = 300) (***P < 0.001 and *P < 0.05).

To confirm the relationship between HOTAIRM1 expression and lung cancer as well as the potential of HOTAIRM1 as a biomarker of lung cancer, we detected the expression level of HOTAIRM1 in the 300 peripheral blood samples collected from patients with lung cancer. The expression level of HOTAIRM1 in the peripheral blood of lung cancer patients was associated with smoking history (*P* = 0.039), lymph node metastasis (*P* = 0.0028), TNM stage (<0.0001), and histological tumor type (*P* = 0.016) (Table 1). In addition, the expression level of HOTAIRM1 was significantly decreased in the peripheral blood of lung cancer patients compared with that of healthy controls. Further analysis revealed that HOTAIRM1 expression levels were the highest in lung ADC compared with small cell lung cancer and squamous cell lung carcinoma (Figures 3A,B). In addition, the expression level of HOTAIRM1 was increased in the peripheral blood of lung cancer patients postoperation, which was contrary to the expression change of Arg1 (Figures 3C,D). This result suggested that HOTAIRM1 might be an indicator of surgical outcomes. In the following correlation analysis, the level of HOTAIRM1 was found to be negatively correlated with both the proportion of MDSCs and the Arg1 level and positively correlated with the proportion of Th1/CTL cells (Figure 3E). The above findings indicate that HOTAIRM1 may be a potential biomarker for differential diagnosis and for the detection of curative effects.

**Table 1:** Correlation between HOTAIRM1 expression and clinicopathological parameters of lung cancer patients (*n* = 300).

Parameter	Number	Relative expression of HOTAIRM1		
		Low	High	*P*-value
Age				0.1651
<60	99	21	78	
≥60	201	60	141	
Gender				0.3729
Male	221	65	156	
Female	79	17	52	
Tumor size (cm)				0.517
≤3	72	48	24	
>3	228	28	200	
Smoking history				0.018
Smokers	197	34	163	
Never smokers	103	50	53	
Lymph node metastasis				0.0095
Positive	202	26	176	
Negative	98	45	53	
TNM stage				0.0013
I + II	37	23	14	
III + IV	263	42	221	
Histological tumor type				0.0231
Squamous cell carcinoma	160	41	119	
Adenocarcinoma	72	18	54	
Small cell lung cancer	68	26	42	

### HOTAIRM1 Enhances the Expression of HOXA1

**Figure 4:**
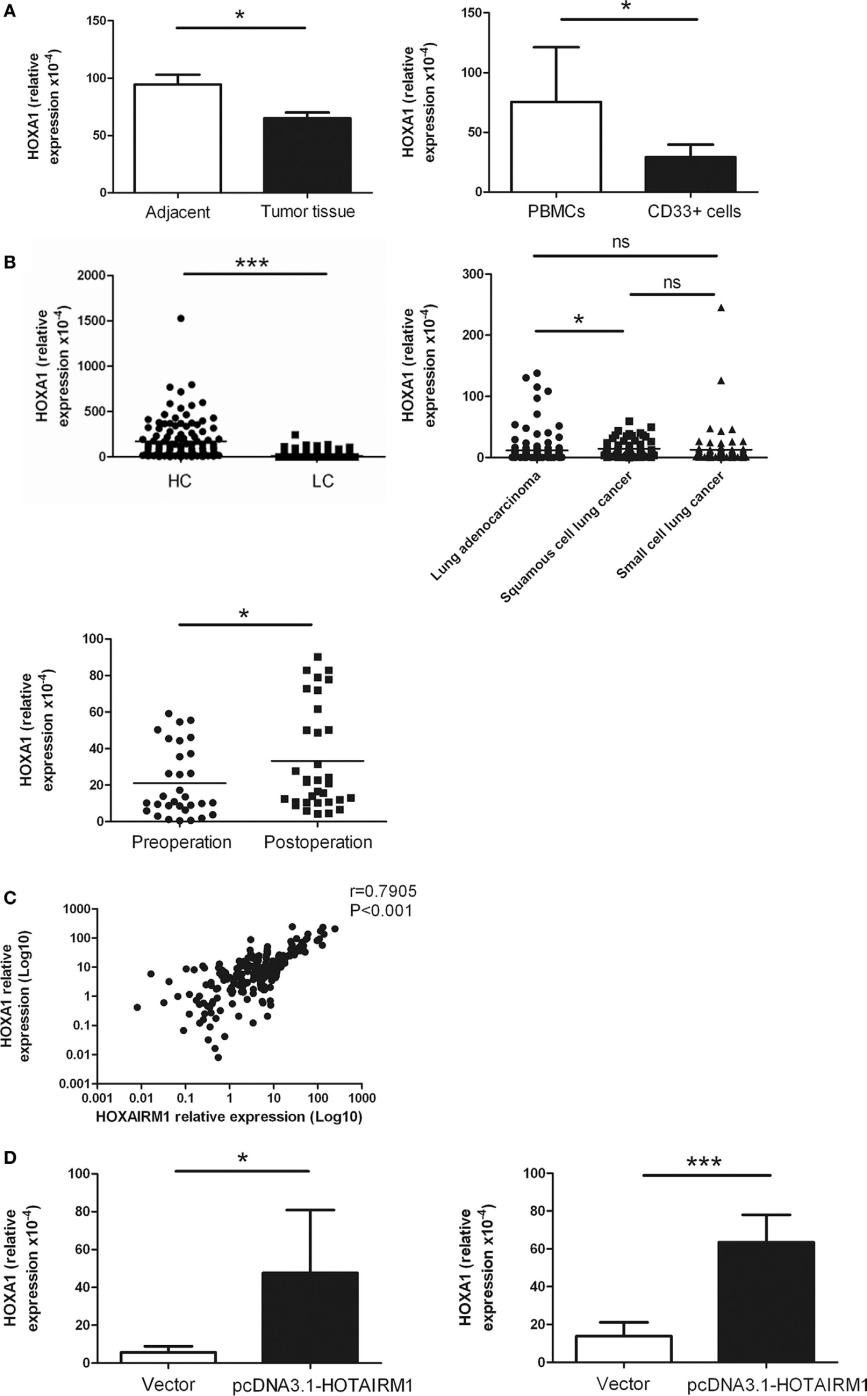
HOXA transcript antisense RNA myeloid-specific 1 (HOTAIRM1) enhances the expression of HOXA1. **(A)** The expression of HOXA1 in myeloid-derived suppressor cells (MDSCs) isolated from tumor tissues of patients with lung cancer (left) or induced from peripheral blood mononuclear cells (PBMCs) of healthy donors with GM-CSF (40 ng/mL) + IL-1β (40 ng/mL) (right) was detected by qRT-PCR. **(B)** HOXA1 levels in the peripheral blood of healthy controls (*n* = 285) and patients with lung cancer (*n* = 300), and the expression of HOXA1 in the peripheral blood of different types of lung cancer (*n* = 300). **(C)** HOXA1 levels in the peripheral blood of lung cancer patients pre- and postoperation (*n* = 42). **(D)** The MDSCs isolated from tumor tissues of patients with lung cancer (left) or induced from PBMCs of healthy donors with GM-CSF (40 ng/mL) + IL-1β (40 ng/mL) (right) were transfected with pcDNA3.1-HOTAIRM1, and HOXA1 levels were detected by qRT-PCR (***P < 0.001, *P < 0.05, and ns, no significance).

HOXA transcript antisense RNA myeloid-specific 1 is an intergenic lncRNA located between HOXA1 and HOXA2. Intergenic lncRNAs have a feature of regulating the expression of adjacent genes. A study had previously demonstrated that HOTAIRM1 regulates the development of granulocytes by enhancing the expression of HOXA1 (29). Here, to confirm whether HOTAIRM1 also mediates the immunosuppression of MDSCs by targeting HOXA1, we first determined whether HOXA1 is expressed in MDSCs. The expression level of HOXA1 was detected in isolated MDSCs from tumor tissues of patients with lung cancer and in induced MDSCs from PBMCs of healthy donors. The results showed that the HOXA1 expression level was decreased in MDSCs from tumor tissues compared with those from adjacent tissue; similarly, the HOXA1 expression level was decreased in induced MDSCs compared with PBMCs (Figure 4A). Detection of HOXA1 levels in peripheral blood confirmed that HOXA1 expression levels were decreased in lung cancer patients compared with healthy controls, and further study indicated that the HOXA1 level was highest in lung ADC compared with small cell lung cancer and squamous cell lung carcinoma. In addition, the expression level of HOXA1 was increased in the peripheral blood of lung cancer patients postoperation (Figure 4B). Moreover, the HOXA1 level in the peripheral blood of lung cancer patients was positively correlated with the expression level of HOTAIRM1 (Figure 4C). Finally, the level of HOXA1 was significantly enhanced when HOTAIRM1 was overexpressed (Figure 4D). These data indicate that HOTAIRM1 can enhance the expression of HOXA1 in MDSCs.

### HOXA1 Is Associated with the Immunosuppression of MDSCs

**Figure 5:**
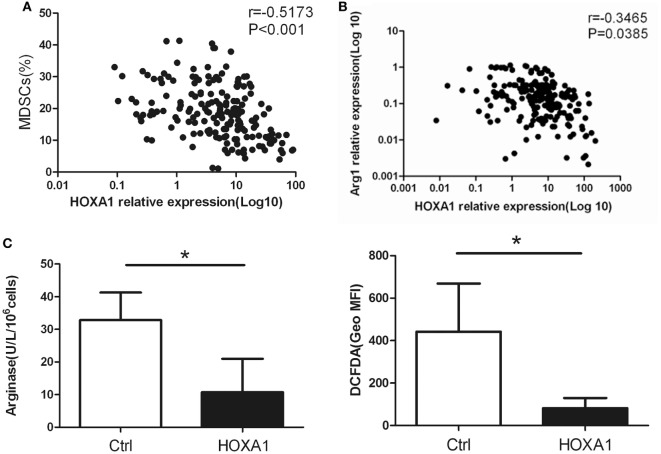
HOXA1 is associated with the immunosuppression of myeloid-derived suppressor cells (MDSCs). **(A)** Correlation analysis between HOXA1 levels and the proportion of MDSCs in the peripheral blood of lung cancer patients (*n* = 300). **(B)** Correlation analysis between HOXA1 levels and arginase 1 (Arg1) levels in the peripheral blood of lung cancer patients (*n* = 300). **(C)** Arg1 activity and reactive oxygen species production in MDSCs from the spleen of tumor-bearing mice following transfection with pcDNA3.1-HOXA1 (*P < 0.05).

Correlation analysis showed that HOXA1 levels were negatively correlated with the proportion of MDSCs and the Arg1 levels in the peripheral blood of patients with lung cancer (Figures 5A,B). To further confirm whether HOXA1 is associated with the immunosuppression of MDSCs, we constructed an HOXA1 expression vector and transfected it into MDSCs isolated from the spleen of tumor-bearing mice. The results showed that HOXA1 overexpression could downregulate Arg1 activity and ROS production in MDSCs (Figure 5C). These data indicate that HOXA1 is associated with immunosuppressive molecules produced by MDSCs.

### Overexpression of HOXA1 Can Delay Tumor Progression and Enhance the Antitumor Immune Response

**Figure 6:**
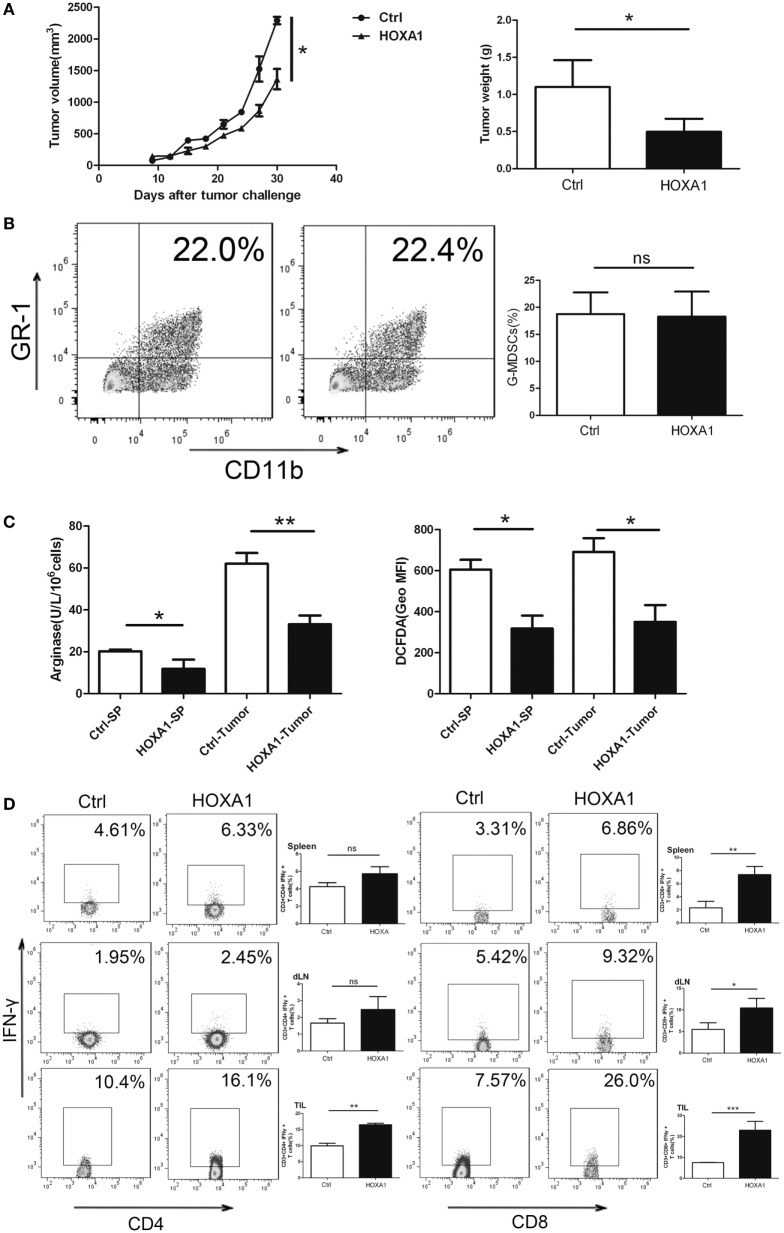
Overexpression of HOXA1 can delay tumor progression and enhance the antitumor immune response in tumor-bearing mice. Groups of mice bearing established LLC cells were intratumorally injected with pcDNA3.1-HOXA1 every 3 days for 3 weeks. **(A)** Tumor volume and weight were measured at the indicated times. **(B)** The proportion of CD11b^+^Gr1^+^ MDSCs in tumor tissue was analyzed by flow cytometry. **(C)** Arginase 1 activity and reactive oxygen species production in MDSCs sorted from the spleen and tumor tissues were detected. **(D)** The proportions of CD3^+^CD4^+^IFN-γ^+^ Th1 and CD3^++^CD8^+^IFN-γ^+^ CTL cells from spleens, draining lymph nodes and tumor tissue were analyzed by flow cytometry (***P < 0.001, **P < 0.01, *P < 0.05, and ns, no significance).

To investigate whether HOXA1 could directly impair the antitumor immune response, we first transplanted 0.9 × 10^6^ LLC cells into C57BL/6 mice. After a palpable tumor was formed, tumor-bearing mice were treated by intratumoural injection of pcDNA3.1-HOXA1 every 3 days for 3 weeks. On the third week, MDSCs from tumor tissues and spleen were isolated, and Arg1 activity and ROS production in MDSCs were measured. As shown in Figures 6A,B, the proportion of MDSCs declined, and tumor progression was delayed. At the same time, Arg1 activity and ROS production were also reduced in MDSCs sorted from tumor tissues and spleen when HOXA1 was overexpressed (Figure 6C). In addition, overexpression of HOXA1 enhanced CD4^+^ Th1 and CD8^+^ CTL cell priming (Figure 6D). These results suggest that overexpression of HOXA1 enhances the antitumor T cell response and delays tumor progression.

## Discussion

Long non-coding RNAs have been identified as crucial regulators of various cells through numerous mechanisms. The specific regulatory mechanism is associated with the localization of lncRNAs in the cell. In the nucleus, lncRNAs can act as a scaffold, recruiting activators or suppressors of transcription to bind to the promoter of target genes. In addition, lncRNAs in the nucleus can epigenetically regulate target gene transcription by inducing histone modifications or chromatin remodeling. In the cytoplasm, lncRNAs act as a sponge for microRNAs (24, 34). However, the role of lncRNAs in MDSC development remains unknown.

In this study, we found that the expression level of the lncRNA HOTAIRM1 was decreased in MDSCs. When confirming the influence of HOTAIRM1 in the development of MDSCs, it was indicated that overexpression of HOTAIRM1 could reduce the immunosuppression of MDSCs and inhibit the induction of MDSCs from PBMCs of healthy donors. Moreover, we found that HOTAIRM1 levels in the peripheral blood of lung cancer patients were significantly decreased compared with those in healthy controls. In a previous study, it was demonstrated that HOTAIRM1 could modulate granulocyte differentiation by targeting HOXA1 (29, 30). Thus, we investigated whether HOTAIRM1 also modulated the development and function of MDSCs by targeting HOXA1. First, we confirmed the expression of HOXA1 in MDSCs; then, we showed that overexpression of HOTAIRM1 could increase the expression of HOXA1 in MDSCs. Similar to HOTAIRM1, HOXA1 expression levels were negatively correlated with the proportion of MDSCs and Arg1 levels and positively correlated with the number of Th1/CTL cells in the peripheral blood of patients with lung cancer. In addition, overexpression of HOXA1 could reduce the immunosuppression of MDSCs and delay tumor progression. Our research revealed that HOTAIRM1/HOXA1 can downregulate the expression of suppressive molecules released by MDSCs and enhance the antitumor immune response.

However, several issues in this investigation remain to be resolved, such as the exact regulatory mechanism of HOTAIRM1/HOXA1 in MDSC function and the factors that induce the downregulation of HOTAIRM1 in MDSCs under tumor conditions. In addition, our results indicated that lncRNAs in circulation may be a biomarker of cancer. Thus, we hypothesize that circulating HOTAIRM1 in the serum of lung cancer patients may be a new marker with which to reflect the progression of lung cancer and the postoperative curative effect. However, these topics require further investigation.

## Ethics Statement

This study was carried out in accordance with the ethical committee of the Affiliated People’s Hospital with Jiangsu University with written informed consent from all subjects. All subjects gave written informed consent in accordance with the Declaration of Helsinki. The protocol was approved by the ethical committee of the Affiliated People’s Hospital with Jiangsu University.

## Author Contributions

XT performed the experiments, analyzed the data, and wrote the paper; JM, JT, HX, and LM analyzed the data; TW and YZ performed the experiments; and SW designed the study and wrote the paper. All the authors have read and approved the final manuscript.

## Conflict of Interest Statement

The authors declare that the research was conducted in the absence of any commercial or financial relationships that could be construed as a potential conflict of interest.
