# Corrigendum: Long Non-Coding RNA HOXA Transcript Antisense RNA Myeloid-Specific 1–HOXA1 Axis Downregulates the Immunosuppressive Activity of Myeloid-Derived Suppressor Cells in Lung Cancer

**DOI:** 10.3389/fimmu.2019.02929

**Published:** 2020-01-21

**Authors:** Xinyu Tian, Jie Ma, Ting Wang, Jie Tian, Yue Zhang, Lingxiang Mao, Huaxi Xu, Shengjun Wang

**Affiliations:** ^1^Department of Laboratory Medicine, The Affiliated People's Hospital, Jiangsu University, Zhenjiang, China; ^2^Department of Immunology, Jiangsu Key Laboratory of Laboratory Medicine, School of Medicine, Jiangsu University, Zhenjiang, China; ^3^Department of Laboratory Medicine, Jiangsu Cancer Hospital, Nanjing, China

**Keywords:** long non-coding RNA, HOXA transcript antisense RNA myeloid-specific 1, HOXA1, myeloid-derived suppressor cells, lung cancer

In the original article, there was a mistake in Table 1 as published. The *p*-values in the table were miscalculated. The corrected Table 1 appears below.

**Table 1:** Correlation between HOTAIRM1 expression and clinicopathological parameters of lung cancer patients (*n* = 300).

**Parameter**	**Number**	**Relative expression of HOTAIRM1**		
		**Low**	**High**	***P*-value**
Age				0.1651
<60	99	21	78	
≥60	201	60	141	
Gender				0.3729
Male	221	65	156	
Female	79	17	52	
Tumor size (cm)				0.517
≤3	72	48	24	
>3	228	28	200	
Smoking history				0.018
Smokers	197	34	163	
Never smokers	103	50	53	
Lymph node metastasis				0.0095
Positive	202	26	176	
Negative	98	45	53	
TNM stage				0.0013
I+II	37	23	14	
III+IV	263	42	221	
Histological tumor type				0.0231
Squamous cell carcinoma	160	41	119	
Adenocarcinoma	72	18	54	
Small cell lung cancer	68	26	42	

The authors apologize for this error and state that this does not change the scientific conclusions of the article in any way. The original article has been updated.

